# Supplementary material for: Learning curve and surgical outcome of robotic assisted colorectal surgery with ERAS program
Source: Sci Rep. 2022 Nov 29;12:20566. doi: 10.1038/s41598-022-24665-w (PMC9709162; doi:10.1038/s41598-022-24665-w)
Supplement: Supplementary file 2 — Supplementary Information 2. [file 41598_2022_24665_MOESM2_ESM.zip › RAL Raw data-Ñ[▒K/063/OP N_ñw╝╨░O▒Kñσ.pdf]

W52 / W52

臺中榮民總醫院
手術室護理記錄

[redacted]

基本資料

- 室別： 開刀房-17室，手術科別： CRS，手術類別： 常規，日期： 2021/03/11
- 進入時間： 0740 ； 麻醉開始時間： 0800 ； Time Out時間： 0909 ； 劃刀時間： 0910 ； 手術結束時間： 1425 送出時間： 1440 ；
- 麻醉方式： GE
  診斷：CA OF RECTUM, L/3
  手術名稱：Robot Assisted Low Anterior Resection
  手術局部用藥：NIL
  術前預防性抗生素：
  - 抗生素名稱一： Veterin 1GM IVD/由麻醉護理師執行
  - 時間： 0850
  - 上次簽章: 何育晴

  ，追加預防性抗生素：
  - 抗生素名稱一： Veterin 1GM IVD/由麻醉護理師執行
  - 時間： 1250
  - 上次簽章: 李郁萱
- 手術醫師： 蔣鋒帆，楊曙榮
- 麻醉醫師： VS 陳永銘
-
  - 刷手護士A ： 李郁萱，時間： 0740，刷手護士B ： 羅媗鈴，時間： 0740，接替刷手一： 廖子晴，時間： 1200，接替刷手二： 林愛甯，時間： 1300
  - 流動護士A ： 何育晴，時間： 0740，接替流動一： 羅媗鈴，時間： 1100，接替流動二： 李郁萱，時間： 1230

皮膚狀況

A-V Shunt： 無
術前： 完整
記錄者： 上次簽章: 何育晴
術後： 完整
記錄者： 上次簽章: 李郁萱

手術準備

手術臥式 單一手術臥式

-
  - 手術臥式： 垂頭仰臥
  - 使用附件： 溫毯 38 ℃，矽膠枕頭，腳架，側身架，其它： silicon pad
  - 皮膚準備： 10%Aq Betadine，Hibiscrub，2% Chlorhexidine
  - 電燒： 使用電燒: 電極片位置： 右大腿，記錄者： 上次簽章: 何育晴

止血帶T： 無

植入物

- 植入物名稱/號碼/數量/部位：Ethicon EEA 33mmx1/Rectum
  davinci endo wrist stapler 45mm green reload x2/Rectum
  Coviden GIA 80-3.8mm*1/Colon

，記錄者： 上次簽章: 李郁萱

標本送檢

- 細菌培養 無
- 細胞學 無
- 冰凍切片 無
- 病理標本
  組織名稱一：Rectum

,
組織名稱二： distal cut end

,
組織名稱三： IMA root

- 截肢 無

標本交付 標本： 3 袋 ，標本： 0 盒 ，標本： 0 瓶 ，細胞學： 0 管 ，Culture Tube： 0 管 ，被交付醫師： CR 楊曙榮，

- 記錄者： 上次簽章: 李郁萱
- 時間： 1400

傷口術後敷料

傷口敷料： 傷口敷料： 無

- 請於圖上標示部位：

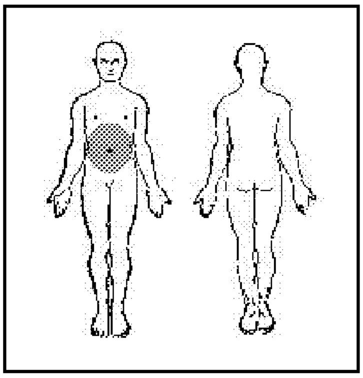

- 傷口敷料：柔軟敷料

導管

導管	天數	導管資訊	插入日期	插入時間	更換日期	拔除日期	拔除時間	拔除原因	預計拔除/更換	預計拔除/更換日期
Double Lumen CVC(1)	1	號碼:7 Fr; 深度:fix15cm; 部位:jugular (右); 象限:RU;	2021/03/11	0835						
Epidural PCA(1)	1	部位:脊髓;	2021/03/11	0815						
Foley(1)	1	號碼:16 Fr.; 部位:膀胱; 註記:ballon:10cc;	2021/03/11	0830						
A-line (1)	1	號碼:20 Ga; 部位:左手;	2021/03/11	0830						

用物計數

- 手術中出血量： 100 c.c.
- 手術中尿量： 400 c.c.
-
    - 其它： 無

用物計數： 多切口同時縫合

- 1.器械： 正確，2.縫針： 正確，3.刀片： 正確，4.紗布： 正確，5.腹部墊： 未使用，6.紗球： 未使用，7.PEANUTS ： 未使用，8.紗條： 未使用，9.絲帶： 未使用，10.針頭： 未使用，11.Vessel loop： 未使用，12.Boots： 未使用，13.Jelco： 未使用，14.棉條： 未使用，
    - 第一次：
        - 刷手護士 ： 廖子晴
        - 流動護士： 上次簽章: 李郁萱
        - 時間： 1320

    ,
    - 第二次：
        - 刷手護士 ： 廖子晴
        - 流動護士： 上次簽章: 李郁萱
        - 時間： 1335

    ,
    - 第三次：

- 刷手護士 ： 廖子晴
- 流動護士： 上次簽章: 李郁萱
- 時間： 1350

備註

備註： 腹腔鏡No.1
達文西Scope NO.1

- 記錄者： 李郁萱 上次簽章: 李郁萱
- 時間： 1440

填寫護理人員 李郁萱

正式摘要

W52 / W52

doctorRank:B
